# Comparison of Match External Loads across a Men’s and Women’s Lacrosse Season

**DOI:** 10.3390/jfmk8030119

**Published:** 2023-08-14

**Authors:** Jennifer B. Fields, Andrew R. Jagim, Nicholas Kuhlman, Mary Kate Feit, Margaret T. Jones

**Affiliations:** 1 Department of Nutritional Sciences, University of Connecticut, Storrs, CT 06269, USA; nicholas.kuhlman@uconn.edu; 2 Department of Exercise Science and Athletic Training, Springfield College, Springfield, MA 01109, USA; mfeit@springfieldcollege.edu; 3 Patriot Performance Laboratory, Frank Pettrone Center for Sports Performance, George Mason University, Fairfax, VA 22030, USA; jagim.andrew@mayo.edu; 4 Sports Medicine Department, Mayo Clinic Health System, La Crosse, WI 54601, USA; 5 Sport, Recreation, and Tourism Management, George Mason University, Fairfax, VA 22030, USA

**Keywords:** GPS, athlete monitoring, workloads, sport science

## Abstract

The purpose of this study was to compare external workloads between collegiate men’s (MLAX) and women’s lacrosse (WLAX) matches and examine positional differences across the season. Athletes (MLAX: *n* = 10; WLAX: *n* = 13) wore a global positional system device during all matches. External load metrics included in the analysis were total distance (TD), sprint distance (SD), accelerations (>3 m/s^2^), sprint efforts, player load per minute (PL/min), top speed, and distances spent in various speed zones. WLAX had higher TD (*p* = 0.001), SD (*p* < 0.001), distances in SZs 2–5 (*p* < 0.001), PL (*p* < 0.001), and sprint efforts (*p* < 0.001) compared to MLAX. However, MLAX performed more acceleration (*p* < 0.001) and deceleration (*p* < 0.001) efforts. WLAX midfielders (M) and defenders (D) reached higher top speeds and performed more accelerations than attackers (*p* < 0.001). Midfielders covered the greatest distance at high speeds (*p* = 0.011) and the smallest distance at low speeds (<0.001) for WLAX. For MLAX, midfielders performed the highest SDs, top speeds, accelerations, decelerations, and distances in higher speed zones (*p* < 0.001) compared to attackers and defenders. Results indicate that there are significant gender and positional differences in external workload demands during match play, specifically for volume- and intensity-derived workload parameters, between men’s and women’s lacrosse. Therefore, sports performance coaches should create gender- and position-specific conditioning programs to prepare athletes for match demands.

## 1. Introduction

Lacrosse is considered one of the most physically demanding team sports due to its high reliance on aerobic and anaerobic fitness, stamina, strength, power, agility, mobility, and sport-specific skills [1,2]. At the collegiate level, matches consist of four 15-min quarters and require quick transitions with abrupt changes in speed and direction and multiple high-intensity sprint efforts up and down the field [3,4]. National Collegiate Athletic Association (NCAA) lacrosse has experienced tremendous growth in recent years (59% increase since 2006), with 191 teams currently competing at the Division (D) I level (men: 74; women: 117), 195 teams at the DII level (men: 81; women: 114), and 540 teams at the DIII level (men: 247; women: 293) [5,6], yet limited information is currently available on the physical demands of collegiate men’s and women’s lacrosse, particularly if differences exist between the two. Of the available research, the majority of studies have reported external workloads in DI women athletes [4,7,8,9,10], despite DIII lacrosse participation accounting for the majority of collegiate programs. With 1–2 matches per week separated by 48–72 h, frequent travel, and further burdens of academic requirements, quantifying workload becomes increasingly important in a collegiate population to help manage the overall stress incurred by athletes [11]. Tracking workloads may be beneficial to enhance recovery, reduce the risk of injury and overtraining throughout the season, and better direct strength and conditioning efforts [12]. Although the physical demands of a competitive lacrosse season are thought to vary between men and women, largely due to match play differences, the respective workload outputs as a function of gender have yet to be evaluated.

Technological advances in athlete tracking have afforded the ability to quantify and monitor external workloads longitudinally, which is important in managing athlete health and sports performance throughout a season. The physical work incurred by an athlete during training, often described as the external load [13], can be quantified using global positioning satellite (GPS) systems [14,15]. External loads commonly collected from GPS devices include volume- (i.e., total distance, PlayerLoad) and intensity- (i.e., sprint efforts, sprint distance, accelerations, decelerations) derived metrics [14,16]. Such technology enables practitioners to gain insights into sport-specific demands, which may inform individualized periodization, recovery, and performance optimization strategies [14,15]. However, in order to individualize the intensities and durations of training sessions, the physical demands of competition must first be identified. Research designed to assess competition workloads has grown rapidly, yet less progress has been made in the sport of lacrosse.

Further, individualized periodization strategies should account for playing position, as workloads are heavily influenced by position-specific demands [7,17,18]. For example, previous studies in elite [19] and collegiate [7,17] men and women lacrosse players have reported higher intensity-dependent movement profiles in midfielders compared to attackers and defenders. While no studies have examined gender differences in seasonal external workloads in collegiate lacrosse, there have been workload similarities shown within collegiate and professional basketball [20], as well as collegiate soccer [11]. However, lacrosse is unique in that match regulations differ across genders. Specifically, collegiate lacrosse is played with 10–12 players on each team (men’s lacrosse (MLAX): one goalkeeper, three defense, three midfielders, three attack; women’s lacrosse (WLAX): one goalkeeper, four defense, three midfielders, four attack) [21]. Consequently, the difference in the number of field players in the men’s and women’s matches may influence differences in workloads performed.

Therefore, the primary aim of this study was to compare match external workloads and positional differences in men and women throughout an entire Division III lacrosse season. A secondary aim was to examine within-sport positional differences. It was hypothesized that external workloads would differ between MLAX and WLAX and among positional groups. This study adds to the existing literature comparing positional differences within their respective sports, but also provides novel insights into gender comparisons of playing position.

## 2. Materials and Methods

### 2.1. Study Design

A retrospective, observational, cohort study design was used. During the 15-week in-season period, all match external loads were measured using GPS technology.

### 2.2. Participants

NCAA DIII men (*n* = 10; age: 18–22 years; body height: 180 ± 6 cm) and women (*n* = 13; age: 18–22 years; body height: 164 ± 6 cm) lacrosse players participated in this study, for a total of 299 player observations. Inclusion criteria were (a) athletes (between the ages of 18–22) who played field positions, and (b) athletes who played more than 50% of each match. Exclusion criteria were (a) athletes who played less than 50% of each match, (b) athletes who were currently injured, and (c) goalkeepers, due to differences in sport activity. Players were further classified by gender and position: defenders (MLAX: *n* = 2, WLAX: *n* = 3), midfielders (MLAX: *n* = 4, WLAX: *n* = 7), and attackers (MLAX: *n* = 4, WLAX: *n* = 3). All players were medically cleared for intercollegiate athletic participation, had the risks and benefits explained to them beforehand, signed an institutionally approved written consent form to participate, and completed a medical history form. This study’s procedures were approved by Springfield College’s Institutional Review Board for the use of human subjects in research (Approval Date: 8 August 2022; IRB# 3452122).

### 2.3. External Load

External load was collected during all matches, which excluded warm-ups (MLAX matches = 16; WLAX matches = 19), throughout the 15-week season using 10 Hz GPS technology (Playertek, Catapult Sports, Melbourne, Australia) [22]. These devices used a minimum of 3 satellites, and units were turned on outside 30 min before training to allow the acquisition of satellite signals and the synchronization of the GPS clock with satellites’ atomic clock [23]. To promote reliability, players wore the same unit for each match/training session throughout the season. Devices were worn according to manufacturer guidelines in a supportive harness positioned between the scapulae. After each match, data were downloaded using the proprietary software, which automatically detected and filtered the data.

External load metrics collected were total distance (TD) (m), sprint distance (SD, >5 m/s), sprint efforts, acceleration efforts (>3 m/s^2^), deceleration efforts (>−3 m/s^2^), top speed, distances in speed zones 1 (SZ1: 0–30% max speed), 2 (SZ2: 30–50% max speed), 3 (SZ3: 50–75% max speed), 4 (SZ4: 75–90% max speed,) and 5 (SZ5: >90% max speed), player load, which is calculated as ∑√ (instantaneous rate of change in acceleration in all 3 orthogonal planes), and player load per minute (PL/min). To determine the maximal speed, players formed a 30 m maximal sprint test during pre-season. The maximal speed was continuously adjusted throughout the season if a player achieved a new higher speed. The use of individualized speed zones has been shown to provide more useful information regarding player velocity, especially when comparing different playing positions [24]. Additionally, the use of individualized speed zones may be more useful when comparing higher speed zones (SZ4 and SZ5) across playing levels to modify zones based on physical abilities [25].

### 2.4. Statistical Analysis

SPSS version 28.0 (IBM, Armonk, NY, USA) was used for all analyses. All non-normally distributed variables were log-transformed. An independent samples t-test assessed gender differences in external loads. Cohen’s d effect sizes were calculated and interpreted as follows: small: 0.2; medium: 0.5; large: >0.8 [26]. Separate multiple analyses of variances (MANOVAs) evaluated (1) a 2 × 3 gender by position interaction in external loads, and (2) within-gender positional differences in external loads. Pairwise comparisons utilizing Bonferroni post-hoc analyses were used when a significant finding was observed (*p* < 0.05). Partial eta^2^ (η^2^) effect sizes were calculated as follows: small: 0.01–0.06; moderate: 0.06–0.14; and large: >0.14 [27].

## 3. Results

Values for match external loads from men’s and women’s lacrosse are provided in Table 1. WLAX covered more TD (*p* = 0.001, d = −0.455), SD (*p* < 0.001, d = −1.149), distances in SZs 2–5 (*p* < 0.001, d = −1.174–1.189), and PL (*p* < 0.001, d = −0.911) and performed more sprint efforts (*p* < 0.001, d = −0.911) compared to MLAX. However, MLAX had a higher number of acceleration (*p* < 0.001, d = 0.462) and deceleration (*p* < 0.001, d = 0.559) efforts (Table 1).

Differences in match external loads across gender and positions are shown in Table 2. For attackers, WLAX athletes covered higher TD (*p* = 0.001, d = 0.951), sprint distance (*p* < 0.001, d = 1.287), distances in SZs 2 (*p* < 0.001, d = 1.147) 3 (*p* < 0.001, d = 3.577), 4 (*p* < 0.001, d = 1.403), and 5 (*p* = 0.023, d = 0.595), sprint efforts (*p* < 0.001, d = 1.102), and PL/min (*p* = 0.006, d = 0.739). MLAX reached higher top speeds (*p* < 0.001, d = 2.605), performed more acceleration efforts (*p* < 0.001, d = 1.278), and covered a greater distance in SZ 1 (*p* < 0.001, d = 1.077).

For midfielders, WLAX athletes had higher sprint distances (*p* < 0.001, d = 0.893), sprint efforts (*p* < 0.001, d = 0.939), and distances in SZs 2 (*p* < 0.001, d = 0.945), 3 (*p* < 0.001, d = 1.031), 4 (*p* < 0.001, d = 0.883), and 5 (*p* = 0.003, d = 0.545). MLAX achieved higher top speeds (*p* < 0.001, d = 1.994), performed more acceleration (*p* < 0.001, d = 0.940) and deceleration efforts (*p* < 0.001, d = 1.351), and covered a greater distance in SZ 1 (*p* < 0.001, d = 2.339).

For defenders, WLAX athletes had higher sprint distances (*p* < 0.001, d = 2.279), sprint efforts (*p* < 0.001, d = 2.228), and distances in SZs 2 (*p* < 0.001, d = 1.977), 3 (*p* < 0.001, d = 3.246), 4 (*p* < 0.001, d = 2.286), and 5 (*p* = 0.041, d = 0.733). MLAX achieved higher top speeds (*p* < 0.001, d = 1.168) and covered a greater distance in SZ 1 (*p* < 0.001, d = 2.882).

**Figure 1:**
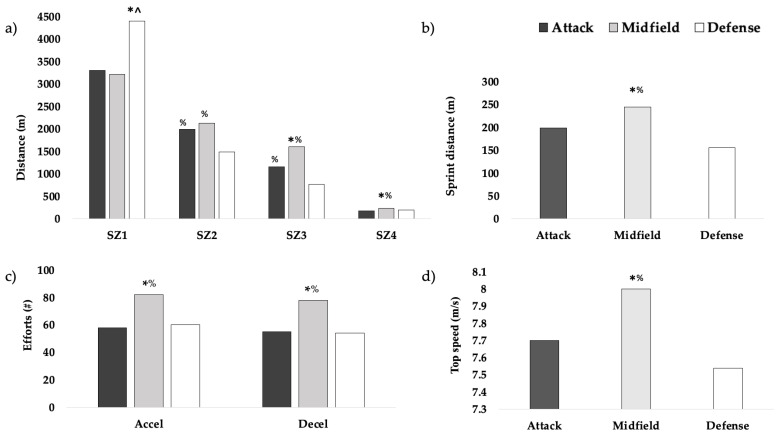
MLAX positional differences in (**a**) distance covered in various speed zones, (**b**) sprint distance, (**c**) sprint efforts, and (**d**) top speed. SZ1: distance covered in speed zone 1; SZ2: distance covered in speed zone 2; SZ3: distance covered in speed zone 3; SZ4: distance covered in speed zone 4; accel: accelerations; decel: decelerations. * higher than attack (*p* < 0.05). ^ higher than midfield (*p* < 0.05). % higher than defense (*p* < 0.05).

For MLAX (Figure 1), significant differences were observed across positions for SD (*p* < 0.001, η^2^ = 0.173), accelerations (*p* < 0.001, η^2^ = 0.292), decelerations, *p* < 0.001, η^2^ = 0.328), sprint efforts (*p* = 0.007, η^2^ = 0.087), distances in speed zones 1–4 (*p* < 0.001, η^2^ = 0.137–0.615), and top speed (*p* < 0.001, η^2^ = 0.132). Specifically, midfielders showed the highest SDs (245 ± 67 m) (*p* < 0.05), top speeds (8.0 ± 0.5 m/s) (*p* < 0.01), accelerations (82 ± 28) (*p* < 0.001), decelerations (78 ± 19) (*p* < 0.001), and distances in higher speed zones (SZ3: 1608 ± 254 m; SZ4: 232 ± 63 m) (*p* < 0.05), compared to attackers and defenders. There were no significant differences across positions for TD (*p* = 0.156), PL (*p* = 0.262), distance in SZ5 (*p* = 0.225), and PL/min (*p* = 0.149).

**Figure 2:**
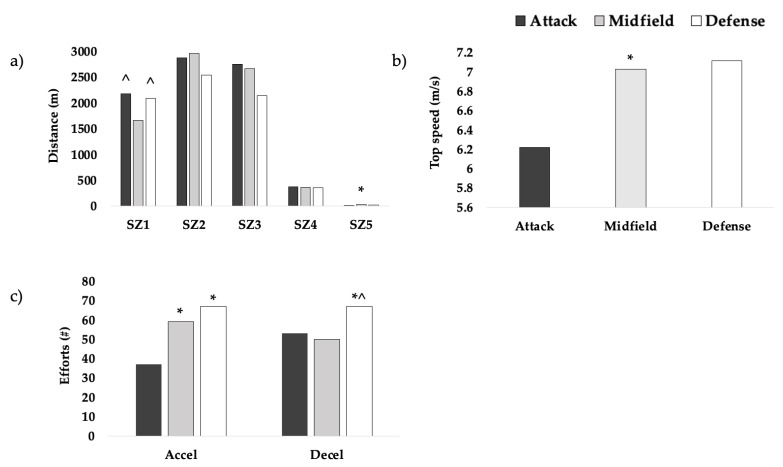
WLAX positional differences in (**a**) total distance, (**b**) top speed, and (**c**) sprint efforts. SZ1: distance covered in speed zone 1; SZ2: distance covered in speed zone 2; SZ3: distance covered in speed zone 3; SZ4: distance covered in speed zone 4; accel: accelerations; decel: decelerations. * higher than attack (*p* < 0.05). ^ higher than midfield (*p* < 0.05).

For WLAX (Figure 2), significant differences were observed across positions for accelerations (*p* < 0.001, η^2^ = 0.129), decelerations (*p* < 0.001, η^2^ = 0.080), top speed (*p* < 0.001, η^2^ = 0.246), and distances in speed zones 1 (*p* < 0.001, η^2^ = 0.098) and 5 (*p* = 0.011, η^2^ = 0.048). Midfielders and defenders reached higher top speeds (M: 7.03 ± 0.49 m/s; D: 7.12 ± 0.33 m/s) (*p* < 0.001) and performed more accelerations (M: 59 ± 21; D: 67 ± 21) (*p* < 0.001) than attackers (top speed: 6.22 ± 0.49 m/s; accelerations: 37 ± 16). Midfielders covered the greatest distance at high speeds (SZ5: 35 ± 45 m) (*p* < 0.05) and the smallest distance at low speeds (SZ1: 1664 ± 591 m) (*p* < 0.01). Defenders performed the greatest number of decelerations (67 ± 24) (*p* < 0.05). There were no significant differences across positions for TD (*p* = 0.299), sprint distance (*p* = 0.840), sprint efforts (*p* = 0.791), or PL (*p* = 0.068).

## 4. Discussion

The primary aim of the current study was to investigate gender differences in external workloads during matches across a competitive season in NCAA DIII men and women lacrosse athletes. These findings are novel in that no prior research has reported differences in seasonal workloads between men and women college lacrosse players, specifically when utilizing the same GPS monitoring system. Further, this study contributes to the limited available literature exploring match demands in DIII athletes, despite the greatest numbers for athlete participation. In addition, the current study explored external loads throughout a full competitive season (15 weeks) and across sport positions, all of which may be used by practitioners to adjust periodization and recovery strategies to optimize athlete health and performance. The main finding of this study indicates that there are significant gender and positional differences in external workload demands during match play, specifically for volume- and intensity-derived workload parameters between men’s and women’s lacrosse.

Findings from the current study indicate that WLAX athletes were exposed to greater TD, SD, sprint efforts, PL, and distances covered at higher speed zones (2–5) compared to MLAX athletes (Table 1). While no other studies have examined gender differences in lacrosse workloads, prior research in collegiate DI WLAX and DIII MLAX is in alignment with the findings from the current study in that WLAX appear to demonstrate greater total distances (~6200–8500 m) [8,18,28], sprint distances (~400–814 m) [7,18,28], and high-intensity sprint efforts (~10–12) [7] than MLAX (TD: ~6000–7000 m; SD: 210–420 m; sprint efforts: 6–14) [17]. However, accelerations (~50) [7], decelerations (~38) [7], and top speeds (6.6–7.2 m/s) [7,8,18] in WLAX do appear lower when comparing prior research to MLAX (accelerations: ~64; top speed: 7.8 m/s), similar to what was observed in the current study. The differences in workloads observed between MLAX and WLAX may be attributed to differences in match regulations and the style of play [21,29]. For example, the higher total distance and sprint metrics observed in WLAX may be attributed to the larger field size (MLAX: 110 × 60 yds; WLAX: 120 × 70 yds). Additionally, the higher sprint metrics may be a result of less physical contact during running movements. Because MLAX permits more physical contacts and impacts, we speculate that MLAX athletes are subsequently moving slower while carrying the ball, which may impede long sprint efforts and subsequently increase the passing frequency. Moreover, due to the higher contact demands involved in MLAX, it may be less likely that these players run straight down the field; they rather engage in more short-cutting movement patterns, which would explain the higher number of accelerations and decelerations when compared to WLAX. Further, the differences in top speed are expected due to differences in type II muscle fiber composition, muscle mass, muscle strength, and muscly quality [30,31].

Only one other study has reported gender differences in lacrosse profiles in elite international MLAX (*n* = 25) and WLAX (*n* = 24) [32]. In agreement with the current study, Weldon et al. [32] reported greater high-intensity acceleration efforts (>4 m/s^2^) and top speeds (~6.2 m/s) in MLAX players; however, they also reported a higher-intensity movement profile in MLAX (distance per minute: 74 m/min; sprint distance: 53 m) [32], which contradicts our findings. The differences in sprint distance observed in the current study are likely due to different speed zone thresholds. For example, Weldon et al. [32] defined the sprint distance as >6 m/s, whereas, in the current study, we defined the sprint distance relative to the maximum velocity (>90% of maximum velocity). Therefore, the current study is unique in that clear comparisons are possible across genders because of the use of the same technological system. Further, the international athletes were playing in the World Lacrosse Sixes, which is a new, shorter match format comprising only six players and a smaller field [33]. Thus, these demands are likely very different from those of a 60-min full-field match and may not be comparable to match play at the collegiate level.

A secondary aim was to examine within-sport positional differences. Interestingly, WLAX attackers covered greater distances and PL/min than MLAX attackers, while these differences were not apparent across the midfield or defense positions. This may be due to match regulations, as WLAX has a longer shot clock (90 s) than MLAX (80 s (20 s to pass midline, then 60 s to shoot)), potentially leading to more ball movement and thus more distance covered. The additional high-speed movements and focus on pushing the ball downfield on offense would also likely explain the higher PL/min observed in WLAX. Additionally, MLAX attackers and midfielders had higher accelerations than WLAX attackers and midfielders, respectively, but no gender difference was observed for defenders.

Monitoring activity profile differences in team sports can provide insights into the requirements of sport-specific positions. The only previous study to explore the match demands of MLAX found that attackers and defenders covered more total distance (attackers: ~7300 m; defenders: ~7200 m), accelerations (attackers: ~80; defenders: ~76), PL (attackers: ~313 AU; defenders: ~294 AU), and PL/min (attackers: 2.91 AU/min; defenders: 2.69 AU/min) than midfielders [17]. However, midfielders covered more of their total distance at higher intensities (sprint distance: ~450 m; sprint efforts: ~14; top speed: 7.8 m/s) [17]. Similar positional differences were observed in the current study, with MLAX midfielders covering the highest sprint distance, top speeds, and distances in higher speed zones, but also performing the greatest number of acceleration and deceleration efforts. This reflects position-specific physical demands, as midfielders have been reported to exhibit the highest level of fitness (i.e., speed and stamina) in lacrosse [3,34,35], likely because they are responsible for covering a larger portion of the field and quickly transitioning the ball back and forth from defense to offense [3,34,35]. As a result, midfielders often engage in more high-intensity efforts compared to other positions; therefore, substitutions may be required more frequently [36]. Midfielders can be further categorized as offensive or defensive players and thus their workloads may vary accordingly; however, the small sample size in the current study prohibited the examination of these differences across sub-groups.

While findings from the current study indicate that distinct positional differences exist in MLAX, such differences are less clear across women lacrosse athletes. For example, several studies have shown no difference in TD, SD, accelerations, and sprints across positions [7,18,28] in NCAA DI players. However, Hauer et al. [37] found that defenders covered the greatest TD and distances at high intensities in international players [37]. While there were no differences across positions in TD, SD, sprint efforts, PL, or PL/min in the current study for WLAX, defenders did achieve the highest maximal speeds (~7.1 m/s), accelerations (~67), and decelerations (~67) [37]. Conversely, Devine et al. [7] reported that DI midfielders had the highest-intensity position profiles, while defenders engaged in the fewest decelerations and sprint metrics due to their location being primarily on one side of the field [7]. Thus, defenders may not have to respond to match demands as frequently as the other positions [7]; however, contextual factors (i.e., level and style of play, tactical strategies, etc.) may influence positional demands and the continued exploration of positional demands is warranted.

Although it is the first study to investigate the gender and positional comparisons throughout an entire NCAA DIII collegiate men’s and women’s season, the current study is not without limitations. For example, the use of different monitoring technologies, (i.e., video analysis, other GPS devices) poses challenges in drawing comparisons across the literature, as each system may provide proprietary metrics to classify match demands or exhibit varying degrees of accuracy, which may limit the generalizability to other programs. It is also important to note that tactical decisions including formation and substitutions may have also influenced match demands. Lastly, data were collected from one NCAA Division III institution in the northeast region and therefore may not be comparable to teams in other divisions or regions. Therefore, future research is warranted that continues to explore these differences across varying levels of competition to aid effective programming strategies.

## 5. Conclusions

In conclusion, monitoring match external workloads can provide coaches with insights to help individualize training programs when preparing athletes for competition. This was the first study to report gender differences in external workloads in collegiate lacrosse players, and the results demonstrated differences in workloads between MLAX and WLAX, as well as across positions. Results suggest that sports performance coaches should create gender- and position-specific conditioning programs to best prepare the athlete for the demands of the sport and position. For example, it may be advantageous to include more sprinting drills for WLAX in preparation for their match demands. Additionally, because WLAX also covered greater distances and PL, conditioning sessions may be of greater importance towards performance and overall fitness development. At the same time, more cutting, changes in direction, and agility drills may be included in MLAX conditioning sessions to prepare for a greater emphasis on accelerations and decelerations and may serve to efficiently prepare them for match movements. In regard to position-specific demands, midfielders from MLAX and WLAX showed the greatest intensity movement patterns during matches compared to other positions, and thus workload monitoring may be crucial to reduce the risk of overtraining and injury.

## Figures and Tables

**Table 1:** Match external loads comparing men’s and women’s lacrosse.

	MLAX	WLAX	*p*-Value	Cohen’s d
TD (m)	6914 ± 1372	7698 ± 2177	<0.001	0.455
SD (m)	210 ± 82	398 ± 213	<0.001	1.149
Accel (# of efforts)	70 ± 26	57 ± 22	<0.001	0.462
Decel (# of efforts)	65 ± 20	52 ± 19	<0.001	0.559
Sprints (# of efforts)	6 ± 3	12 ± 7	<0.001	1.129
PL (AU)	291 ± 55	370 ± 105	<0.001	0.911
SZ1 (m)	3478 ± 1019	1773 ± 645	<0.001	1.990
SZ2 (m)	1953 ± 631	2903 ± 973	<0.001	1.182
SZ3 (m)	1271 ± 403	2622 ± 934	<0.001	1.189
SZ4 (m)	196 ± 78	367 ± 190	<0.001	1.174
SZ5 (m)	14 ± 13	30 ± 42	<0.001	0.488
PL/min (AU/min)	2.66 ± 0.41	2.78 ± 0.61	0.057	0.208
Top speed (m/s)	7.78 ± 0.53	6.93 ± 0.54	<0.001	1.538

Values are Mean ± Std Dev. TD: total distance; SD: sprint distance; accel: accelerations; decel: decelerations; PL: player load; SZ1: distance in speed zone 1; SZ2: distance in speed zone 2; SZ3: distance in speed zone 3; SZ4: distance in speed zone 4; SZ5: distance in speed zone 5. # represents the number of efforts.

**Table 2:** Differences in workloads across gender and positions.

	Attack		Midfield		Defense	
	MLAX	WLAX	MLAX	WLAX	MLAX	WLAX
TD (m)	6655 ± 1526	8190 ± 2098 *	7205 ± 1077	7693 ± 2204	6817 ± 1431	7153 ± 1854
SD (m)	199 ± 82	384 ± 238 *	245 ± 73	402 ± 217 *	154 ± 70	378 ± 140 *
Accel (# of efforts)	58 ± 17	37 ± 16 *	86 ± 31	59 ± 21 *	59 ± 15	67 ± 21
Decel (# of efforts)	55 ± 17	53 ± 13	78 ± 22	50 ± 18 *	54 ± 13	67 ± 24
SZ1 (m)	3617 ± 1553	2386 ± 761 *	3521 ± 769	1820 ± 591 *	4814 ± 948	2289 ± 638 *
SZ2 (m)	2178 ± 585	3145 ± 870 *	2329 ± 756	3235 ± 1019 *	2136 ± 413	2778 ± 651 *
SZ3 (m)	1267 ± 277	3005 ± 648 *	1759 ± 372	2919 ± 987 *	844 ± 235	2341 ± 587 *
SZ4 (m)	200 ± 76	412 ± 230 *	254 ± 70	403 ± 191 *	158 ± 65	390 ± 127 *
SZ5 (m)	18 ± 15	8 ± 11 *	15 ± 11	38 ± 45 *	11 ± 10	23 ± 22 *
Sprints (# of efforts)	6 ± 3	12 ± 8 *	7 ± 2	13 ± 8 *	4 ± 2	12 ± 4 *
PL/min (AU/min)	2.7 ± 0.3	3.0 ± 0.5 *	2.7 ± 0.4	2.8 ± 0.6	2.5 ± 0.7	2.5 ± 0.5
Top Speed (m/s)	7.6 ± 0.6	6.21 ± 0.5 *	8.0 ± 0.5	7.0 ± 0.5 *	7.6 ± 0.4	7.1 ± 0.3 *

Values are Mean ± Std Dev. * *p* < 0.05. TD: total distance; SD: sprint distance; accel: accelerations; decel: decelerations; PL: player load; SZ1: distance in speed zone 1; SZ2: distance in speed zone 2; SZ3: distance in speed zone 3; SZ4: distance in speed zone 4; SZ5: distance in speed zone 5. # represents the number of efforts.

## Data Availability

The data that support the findings of this study are available from the corresponding author upon reasonable request.
